# Assessing Syringe Exchange Program Access among Persons Who Inject Drugs (PWID) in the District of Columbia

**DOI:** 10.1007/s11524-015-0018-5

**Published:** 2016-01-19

**Authors:** Sean T. Allen, Monica S. Ruiz, Jeff Jones

**Affiliations:** Department of Prevention & Community Health, Milken Institute School of Public Health at The George Washington University, 950 New Hampshire Ave Suite 300, Washington, DC 20052 USA; Department of Health Policy & Management, Jiann-Ping Hsu College of Public Health at Georgia Southern University, PO Box 8015, Statesboro, GA 30460 USA; Johns Hopkins University, 615 N. Wolfe St., Room E6534, Baltimore, MD 21205 USA

**Keywords:** Persons who inject drugs, HIV, Syringe exchange programs, Substance use, Access

## Abstract

Prior research has explored spatial access to syringe exchange programs (SEPs) among persons who inject drugs (PWID), but these studies have been based on limited data from short periods of time. No research has explored changes in spatial access to SEPs among PWID longitudinally. The purpose of this research is to examine spatial access to SEPs among PWID who accessed services at a SEP in Washington, District of Columbia (DC), from 1996 to 2010. The geometric point distance estimation technique was used to calculate the mean walking distance PWID traveled from the centroid point of their zip code of home residence to the mobile exchange site where they accessed SEP services. Analysis of variance (ANOVA) was used to examine differences in walking distance measures by year. The results of this research suggest that the distance DC PWID traveled to access SEP services remained relatively constant (approximately 2.75 mi) from 2003 to 2008, but increased to just over 4 mi in 2010. This research provides support for expanding SEP operations such that PWID have increased access to their services. Increasing SEP accessibility may help resolve unmet needs among injectors.

## Introduction

Research has documented the public health utility of syringe exchange programs (SEPs) for persons who inject drugs (PWID). SEPs are cost-effective, decrease the incidence of HIV among injectors, and have not been shown to increase drug use, crime, or presence of discarded syringes in neighborhoods.^1^^–^5 Beyond the provision of sterile injection equipment, PWID may experience other benefits while engaging with SEPs; for example, these programs may provide referrals to other essential health and human services (e.g., substance use treatment programs, basic medical care, etc.) that may help address unmet needs and facilitate substance use cessation.^6^^,^^7^ In order for SEPs to be efficacious, their services must be accessible to the PWID population. Existing literature has shown that PWID who reside greater than 1 mi from a SEP are more likely to have injected with a used syringe in the prior 6 months.^8^ Research has also documented that persons who live within a 10-min walk to SEPs are nearly three times more likely to consistently access services than their counterparts who live further away.^9^

SEP accessibility among injectors may be affected by structural and temporal barriers; for example, in the District of Columbia (DC), a policy (§48-1121) was passed in 2000 prohibiting the distribution of “any needle or syringe for the hypodermic injection of any illegal drug in any area of the District of Columbia which is within 1000 feet of a public or private elementary or secondary school (including a public charter school).” Although no research has empirically examined this policy in depth, it is possible that it restricts SEP operations in areas of greatest need for syringe exchange services and forces PWID to travel greater distances to engage with the SEP. Research has also identified limited hours of SEP operations as a contributing factor to increased likelihood of syringe sharing.^10^

A study quantified the distances between SEPs and areas of relevance to PWID (such as where substances are purchased/used and home residence); however, these findings were based on data from 2002 to 2006 among the Philadelphia PWID population.^11^ According to a recent study that examined SEP access among the PWID population in the DC, active injectors traveled nearly 3 mi (on average) to access SEP services in 2014.^7^ Notably, this study of DC injectors operationalized SEP access as the distance PWID walked via sidewalks to engage with the SEP. Though these studies offer valuable insights into SEP accessibility, they do not examine how SEP access may change over time. This is an important gap in the literature given that SEP accessibility may be a contributing factor for consistent utilization of sterile injection equipment. The purpose of this research is to expand our knowledge of SEP accessibility among the DC PWID population by examining the distance PWID traveled to engage with Prevention Works, the city’s first SEP, from its implementation in 1996 to its closing in 2011. We hypothesized that the average distance persons traveled from their home residence to the SEP would decrease over time as the efficiency of the SEP increased.

## Methods

Exchange records from Prevention Works were used to estimate the walking distance PWID traveled to access syringe exchange services from its implementation to its closing. At the time of registration with the SEP, each client was assigned a unique identifier and asked questions pertaining to race/ethnicity, age, substance use profile, HIV status, and other sociodemographic measures. The unique identifiers were used by the SEP to track the services each PWID accessed, how many syringes they returned/were given, and where they accessed the SEP at each exchange event.

All exchange sites (public parks, intersections, shopping center parking lots, etc.) were geocoded using Google Maps.^12^ Because home residence data were limited to the zip code of residence, the geometric point distance estimation technique was used to estimate the walking distance PWID traveled to access the SEP. In this technique, distance measures are calculated using the geometric centroid (i.e., the geometric center) of a given unit of analysis, such as a zip code or neighborhood, with the assumption that all data pertaining to the unit of analysis have a common origin at the centroid point.^13^^–^15 This methodological approach was used in a previous study that examined differences in SEP access between active and non-active injectors.^7^ Map data of zip codes in the USA were downloaded from the United States Census Bureau^16^ and imported to ArcMap v10.2.1. ArcMap was used to calculate the latitude and longitude coordinates of the centroid point of each zip code.

Because we sought to understand SEP access among DC PWID, any instances where a person reported residing in a zip code outside of the District (thus indicating a non-DC resident) or at a post office box zip code (which may represent a location of convenience rather than a space near the participant’s home) were excluded from the analysis. A SAS macro was used to quantify the walking distance (via sidewalks) between the centroid point of zip code of home residence and SEP exchange site. The walking distance measures were then analyzed by calendar year. Analysis of variance (ANOVA) was used to test for differences between the mean walking distance measures by year. Because the SEP closed in February 2011, data were only analyzed for years in which a complete calendar year of data was available (1996–2010). The George Washington University Institutional Review Board approved this study (IRB# 111421).

## Results

The Prevention Works database included records for 12,094 unique PWID who accessed SEP services from 1996 to 2011. In total, these persons engaged in 77,221 exchanges. The number of exchanges that took place annually ranged from 485 to 14,285. To better understand SEP access among DC PWID and to make the analyses more generalizable, a number of exclusion criteria were applied. The final analytic sample consisted of African-American/Black or Caucasian/White DC PWID who reported being male or female and who reported residing at a geographic zip code (rather than a post office box zip code).

Among the 12,094 persons who engaged with the SEP, 54.3 % (*n* = 6571) had registration data that included information pertaining to zip code of home residence. Chi-square and *t* test analyses were used to compare PWID with and without zip code of home residence data. Those who had home residence data were slightly younger than those without residence data (41.5 and 42.7 years, respectively; *p* < .05). A greater proportion of those with residence data identified as men compared to those with missing residence data (73.1 and 68.4 %, respectively; *p* < .001). Similarly, there were also differences between those with and without residence data in terms of the proportion of PWID that identified as African-American/Black (93.4 and 83.2 %, respectively; *p* < .001). Although these differences were significant, they are most likely reflective of inconsistent data collection at the time PWID registered with the SEP. When persons presented for services, SEP personnel may have focused more on the provision of sterile injection equipment and referrals to other services rather than uniform and complete data collection.

Among those SEP clients with zip code of home residence data (*n* = 6571), a minority of persons were excluded due to small sample sizes. For instance, 0.2 % (*n* = 15) were excluded due to persons identifying as transgender. Transgender clients were also excluded from the analytic sample as it is possible they accessed SEP services to obtain injection equipment for injecting hormones or silicone rather than injecting drugs. An additional 104 persons (1.6 %) were excluded due to missing gender data, leaving 6452 PWID in the preliminary analytic sample. Persons who identified as a race/ethnicity other than African-American/Black or Caucasian/White were also excluded. This resulted in the exclusion of a small number of PWID (*n* = 84); more specifically, it resulted in the exclusion of *n* = 33 persons who identified as Hispanic, *n* = 9 persons who identified as Asian, and *n* = 12 persons who identified as “other” (but without any further information describing what constituted “other”). Other persons (*n* = 30) were excluded from the analyses due to missing race/ethnicity data. These exclusions left 6368 PWID in the preliminary analytic sample who identified as male or female and African-American/Black or Caucasian/White.

The remaining PWID (*n* = 6368) in the sample reported zip codes of home residence in seven different states (MD, VA, CA, MI, NE, CT, and LA) and DC, although the majority (87.8 %, *n* = 5593) reported a DC zip code. Among those that reported a non-DC zip code (*n* = 775), the majority (74.2 %, *n* = 575) reported a MD zip code. Of these 575 persons from a MD zip code, the majority (89.7 %, *n* = 516) reported a zip code in a county adjacent to DC, and these persons were dispersed across 18 different zip codes in that county. Twenty-three percent (*n* = 179) of the PWID outside DC reported a zip code in Virginia, and, similarly, they were geographically dispersed across 15 different zip codes that varied substantially in size and proximity to DC. Among the other five states represented in the data, no state had more than 11 persons reporting a zip code of residence in the given state. To tailor the analyses to DC PWID, only data from persons who reported a DC zip code were included. This yielded 5593 PWID clients from zip codes (of any type) in the District. These persons collectively engaged in 45,899 exchanges.

Among the remaining PWID, 76.9 % (*n* = 4300) had exchange location data that was viable for geocoding for use in the distance estimation calculations (i.e., the location(s) of the exchanges were able to be matched to a specific location). The registration data that were not viable for geocoding stemmed from limitations in how data were recorded in the Prevention Works dataset. More specifically, the exchange locations that were coded as “Various Sites” or “Unidentified” were not able to be geocoded to a specific location and resulted in some persons having no viable exchange data for the purposes of this study. Of the remaining 4300 PWID, 84.6 % (*n* = 3638) reported a zip code of home residence in DC that was a geographical zip code (i.e., not a post office box zip code). These 3638 persons and their collective 33,959 total exchange records formed the analytic sample for these analyses.

The majority of the analytic sample identified as male (74.7 %) and African-American/Black (96.6 %). The mean age at time of registration was 41.0 ± 8.8 years (range 10–73 years). The majority also reported being marginally housed (62.4 %), unemployed (75.2 %), and having completed high school, but not attended college (53.5 %). Slightly more than half of the sample (50.2 %) reported having ever engaged in a drug treatment program. In terms of substance use, the majority reported using heroin (87.6 %) and speedball (56.5 %). These data are summarized in Tables 1 and 2.

**Table 1:** Descriptive statistics of the analytic sample (*n* = 3638)

Variable		% of sample
Gender	Male	74.7
	Female	25.3
Race	African-American/Black	96.6
	White	3.4
Housing status	Not marginally housed	27.4
	Marginally housed	62.4
	Missing housing data	10.2
Engagement in a drug treatment program	Never in a drug treatment program	49.8
	Previously in a drug treatment program	50.2
Employment status	Unemployed	75.2
	Employed part-time	4.6
	Employed full-time	9.6
	Missing employment data	10.5
Education level	Did not graduate high school	23.5
	Graduated high school (no college)	53.5
	Graduated high school (some college, no degree)	9.5
	Graduated from college	3.2
	Missing education data	10.3

**Table 2:** Analytic sample substance use measures (*n* = 3638)

Substance	Reported use	% of sample
Heroin	Did not report use	12.4
	Reported use	87.6
Skin popping	Did not report use	57.5
	Reported use	42.5
Cocaine	Did not report use	66.1
	Reported use	34.0
Speedball	Did not report use	43.5
	Reported use	56.5

**Fig. 1:**
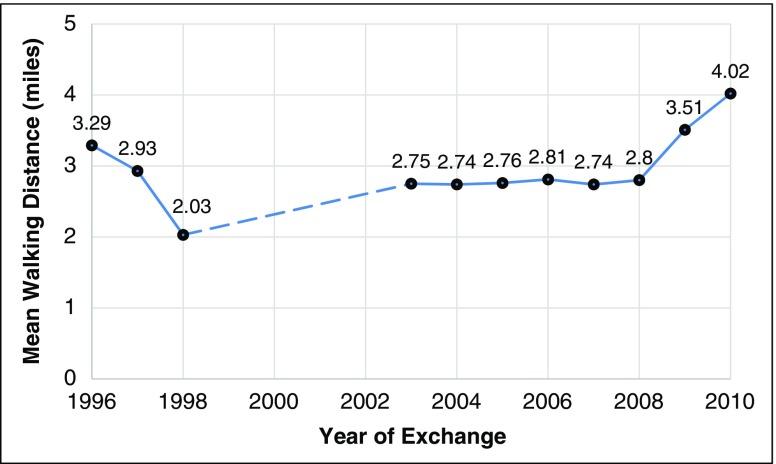
Mean walking distance by year

The SEP dataset did not include viable exchange data that could be used in the distance estimations from 1999 to 2002; however, from 1996 to 1998, the average walking distance between the centroid point of zip code of home residence and exchange site decreased from 3.29 to 2.03 mi (*p* < .05). There were no statistically significant differences in the mean walking distance measures from 2003 to 2008. During these years, the distance measure remained nearly constant, averaging around 2.75 mi. Notably, the mean walking distance increased in the last two complete years the SEP was open (2009 and 2010). More specifically, PWID reported traveling the furthest distances during these years (3.51 and 4.02 mi, respectively). Notably, the estimated mean walking distance in 2010 was significantly (*p* < .05) greater than all preceding year mean estimates. These data are summarized in Tables 3 and 4 and depicted in Fig. 1.

**Table 3:** Walking distance measures by year of exchange

Year of exchange	Viable number of exchanges	Mean walking distance (mi)	Standard deviation	Range
1996	1105	3.29	1.70	8.90
1997	3287	2.93	1.66	9.00
1998	1649	2.03	1.81	9.00
1999	0	0.00	0.00	0.00
2000	0	0.00	0.00	0.00
2001	0	0.00	0.00	0.00
2002	0	0.00	0.00	0.00
2003	2129	2.75	1.94	10.20
2004	7062	2.74	1.96	11.70
2005	5545	2.76	1.99	11.20
2006	4320	2.81	2.03	11.20
2007	3927	2.74	2.01	11.20
2008	2602	2.80	2.09	11.20
2009	836	3.51	2.21	9.60
2010	1497	4.02	2.24	11.20

**Table 4:** ANOVA comparisons of mean walking distance measures by year

Year	1996	1997	1998	2003	2004	2005	2006	2007	2008	2009	2010
1996											
1997	<.0001										
1998	<.0001	<.0001									
2003	<.0001	0.0332	<.0001								
2004	<.0001	0.0003	<.0001	1							
2005	<.0001	0.0028	<.0001	1	1						
2006	<.0001	0.1693	<.0001	0.9947	0.9088	0.9897					
2007	<.0001	0.0012	<.0001	1	1	1	0.9038				
2008	<.0001	0.246	<.0001	0.9995	0.992	0.9996	1	0.9863			
2009	0.376	<.0001	<.0001	<.0001	<.0001	<.0001	<.0001	<.0001	<.0001		
2010	<.0001	<.0001	<.0001	<.0001	<.0001	<.0001	<.0001	<.0001	<.0001	<.0001

## Discussion

These data provide limited support for the hypothesis that the average walking distance PWID traveled to engage with SEPs would decrease over time. The estimated mean walking distance by year decreased during the first few years of Prevention Works’ operations then remained relatively stationary (approximately 2.75 mi) from 2003 to 2008, but later increased to just over 4 mi in 2010. This finding may be explained by the closing of Prevention Works: as the organization began experiencing management and personnel problems, its service delivery became less efficient and/or unpredictable. In other words, the geographic diversity and operational hours of the SEP exchange sites may have changed/decreased and caused PWID to travel greater distances to access services.

As noted previously, a policy implemented in 2000 also exists in the District that prohibits SEPs from operating within 1000 ft of a school. It is plausible that the amount of legal SEP operational space changed over time and resulted in PWID traveling greater distances to access services. Given that there were no viable data for the 1999–2002 period, our ability to extrapolate the possible effects of this policy restriction is limited. Future work should be conducted to examine this policy restriction more in-depth and its possible effects on SEP accessibility. Work should also be conducted among the DC PWID population to understand the possible reasons that drove the shifts in commuting distance.

The results of this study align with similar measures of SEP access among PWID in Philadelphia, PA. According to Williams and Metzger, the approximate distance from home residence to SEP site was approximately 2.5 mi for the Philadelphia PWID population who accessed SEP services from 2002 to 2006.^11^ There were methodological differences between the study of Philadelphia PWID and this research due to limitations in the available data, but the fact remains that the approximate walking distance measures were comparable between the two cities’ PWID populations. Our findings also align with another DC-based study that found that injectors were traveling approximately 2.75 mi to access SEP services in 2014.^7^ Together, this collective evidence addresses an important issue in the public health literature by demonstrating that shifts in SEP access occurred in PWID populations and that these shifts may be due to organization issues (e.g., changes in SEP operations) rather than factors associated with PWID themselves. Future work should explore the relationship between SEP accessibility and exchange patterns among PWID.

The results of this study have a number of limitations that warrant discussion. Foremost, the geometric point distance estimation method assumes that all persons who report a given unit of analysis reside at a geometric centroid point. As noted in a previous study of injectors’ access to SEP services that used this method, this limitation is important “given that gentrification and changes in residential and commercial zoning may lead to uneven housing opportunities and population distributions within a given zip code.”^7^ Secondarily, this study assessed walking distance between zip code of home residence and exchange site. In actuality, it may be the case that other points of interest are more salient in determining SEP utilization (e.g., distance between location of drug purchase and SEP exchange site).

Another limitation of this research is that walking distance was measured rather than distance via automobile or public transportation. Although these modes of transportation may be used by some PWID who access SEP services, it is more likely that majority of DC PWID access services via walking due to the financial burdens associated with private and public transportation.^7^ Future work should explore what mode(s) of transit PWID most commonly use to access services and what routes they take to reach the exchange site.

An additional limitation is that we were unable to determine the extent to which PWID accessed sterile injection equipment via over-the-counter (OTC) purchase at pharmacies. Research has shown that greater spatial access to SEPs and OTC syringe purchase at pharmacies improved injectors’ capacity to engage in harm reduction practices.^17^ However, research has also found that the prevalence of OTC syringe purchase is 53 % lower among African-American PWID.^18^ Given that persons reported traveling greater distances in later years of the study period and that the majority of our analytic sample identified as African-American, important next steps for this research are to examine the prevalence of OTC syringe purchasing among DC PWID and to evaluate differences in the risk profiles of PWID who only access sterile injection equipment at SEPs and those who acquire syringes at both SEPs and pharmacies.

Lastly, data limitations forced these analyses to be based on the collective exchange records of only a portion of the Prevention Works dataset. Given the brief time that PWID engage with the SEP at each exchange event, it is logical that there was a relatively large amount of data that were not viable for these analyses. It is highly likely that when the SEP was in operations, they focused their efforts on distributing sterile injection equipment and referrals rather than ensuring completeness of data collection. Despite these limitations, this study examines the collective exchange activities of more than 3600 PWID over more than a decade and contributes to our understanding of SEP accessibility.
